# Spontaneous rupture of a giant pyonephrosis: A case report

**DOI:** 10.1016/j.radcr.2022.01.056

**Published:** 2022-02-04

**Authors:** Fallou Galass Niang, Ibrahima Faye, Abdourahmane Ndong, Issa Thiam, Abdoulaye Ndoye Diop

**Affiliations:** a Department of Radiology, Saint-Louis Hospital, Gaston Berger University, 2 Ngallele road, Saint-Louis, Senegal; b Department of Surgery, Saint-Louis Hospital, Saint-Louis, Senegal

**Keywords:** Pyonephrosis, Urinary tract infection, Urinary lithiasis, Computed tomography

## Abstract

Pyonephrosis is a suppurative infection of the kidney caused by ureteral obstruction. It can lead to kidney failure, septic shock, and death. Thus, it requires prompt assessment and appropriate management. We report a case of a 63-year-old male with giant pyonephrosis contained 10 liters of pus and spontaneously ruptured in the adjacent muscles. This clinical case illustrates the value of computed tomography scan in the diagnosis and management of an uncommon upper urinary tract infection and its complications.

## Introduction

Pyonephrosis is a rare and severe urinary infection caused by high urinary tract obstruction. It can result in permanent impairment of renal function, septic shock, or death if it left undiagnosed and untreated [1]. Pyonephrosis can be voluminous when the diagnosis is not made at an early stage, and the occurrence of rupture is possible. We report case of spontaneously ruptured giant pyonephrosis with abscess of adjacent muscles in an elderly man.

## Case report

**Fig. 1:**
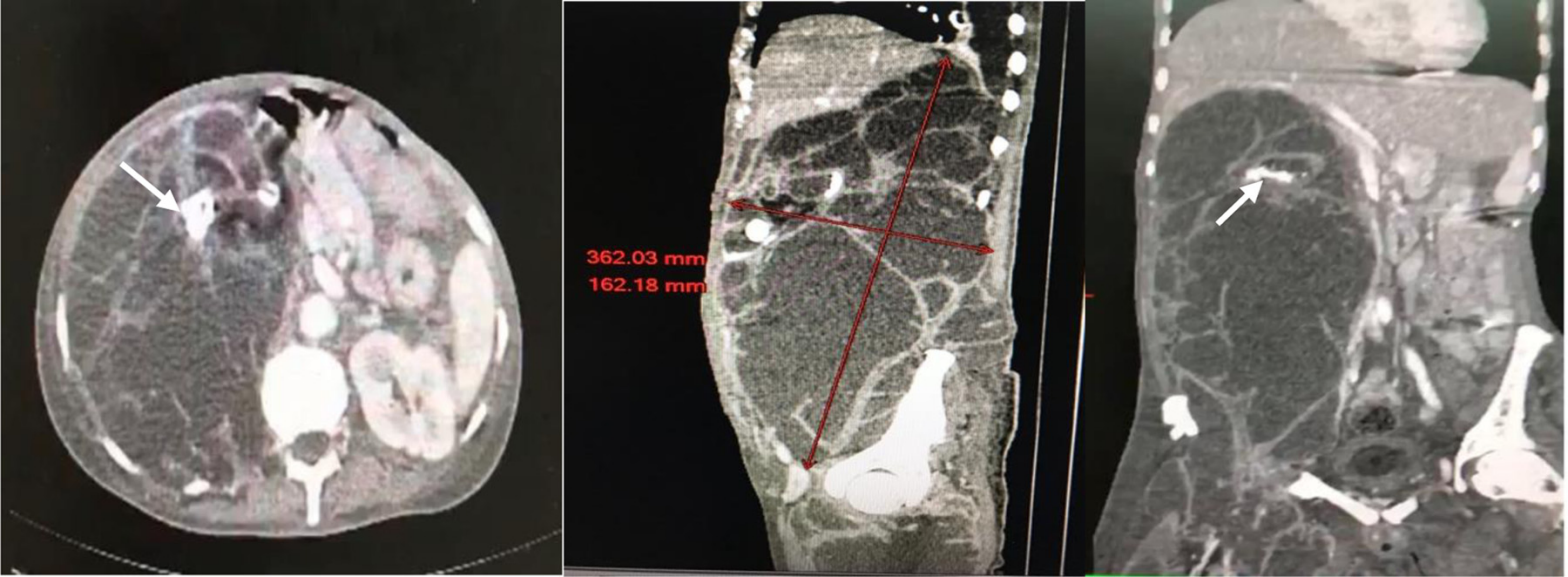
Abdominal and pelvic enhanced-contrast CT: transverse section (A), sagittal (B) and coronal (C) reconstructions showing a large collection extending from the right hypochondrium to the pelvis with enhanced septa and stones (withe arrow) without any visible renal parenchyma.

**Fig. 2:**
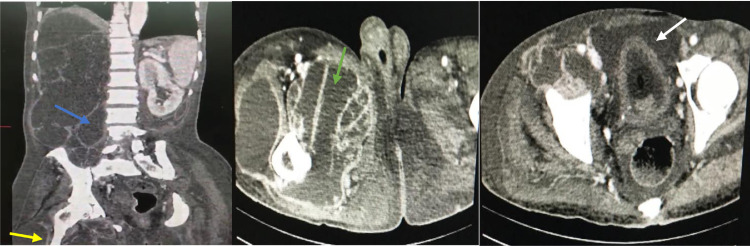
Abdominal and pelvic enhanced-contrast CT: sagittal reconstruction (A) and in transverse sections (B and C) showing collections with the same density as that described above in the right iliac psoas (blue arrows), the right gluteal (yellow arrow) and adductors (green arrow) muscles with low pelvic peritoneal effusion (white arrow).

A 63-year-old patient without any medical history received for abdominal distension with right flank pain, and fever evolving for 2 weeks. Clinical examination revealed an infectious syndrome, abdominal tenderness with a right flank mass. In addition, there was an inflammatory swelling of the right thigh. Abdominal and pelvic contrast-enhanced CT showed an extensive collection with thin septa, extending from the right hypochondrium to the pelvis, measuring approximately 362 mm in height, 120 mm in transverse diameter and 162 mm in anteroposterior diameter (Fig. 1). The right renal parenchyma was no longer detectable, but stones formations were noted in the renal compartment (Fig. 1). There was also a collection with the same density in the iliac psoas muscle, the gluteal muscles and the rectus adductors. Furthermore, there was a peritoneal fluid effusion in the pelvis (Fig. 2). The ultrasound-guided puncture of the collection in the thigh showed pus. A final diagnosis of right sided ruptured giant pyonephrosis with destructed kidney due to ureteropelvic junction obstruction was made. An exploratory laparotomy was performed and confirmed the diagnosis. After aspiration of 10 liters of pus, a nephrectomy was realized. The post-operative course was uneventful.

## Discussion

Pyonephrosis is a suppurative infection of the upper urinary tract and differs from infected hydronephrosis by the presence of pus. It is usually associated with parenchymal damage, chronic pyelonephritis and subsequent loss of kidney function [2]. The most common cause is kidney stones; and they have been found in more than 70% of pyonephrosis cases [3]. In this case, we noted several stones projecting into the right renal compartment, probably responsible for the urinary tract obstruction.

The most common clinical presentation is flank pain with fever and chills. Other possible symptoms are nausea, vomiting, back pain, abdominal distension and nephromegaly [3,4].

Imaging is very essential for the diagnosis and management particularly sonography and computed tomography. Indeed, CT is very sensitive for diagnosis of pyonephrosis and its complications. Moreover, it helps assess renal function, and the perirenal environment looking for perinephric abscess [[5], [6], [7]]. CT can also help to differentiate pyonephrosis from infected hydronephrosis. In fact, in infected hydronephrosis the renal parenchyma does not present suppurative destructive lesions and there is no perinephritis. In addition, it allows to rule out etiologies, particularly abdominal lesions, including metastatic cancer, retroperitoneal fibrosis, and renal stones that are not visible on the ultrasound [2].

Giant pyonephrosis has an exceptional anatomical presentation. Rare authors describe huge lesions measuring around 30 cm in the longest axis [1,8,9]. Rupture of pyonephrosis is also very uncommon. The particularity of this observation was related to the rupture in the adjacent muscles (iliac psoas), with extension to the gluteal and crural muscles. The best treatment remains nephrostomy or nephrectomy [2]. In this case, the treatment was an aspiration followed by a nephrectomy by laparotomy with good outcomes.

## Conclusion

Giant pyonephrosis is a rare suppurative infection of the urinary system. CT plays a key role in the management by determining the etiology and the associated complications. The rupture in the adjacent muscles is an exceptional complication.

## Patients consent

Have been obtained.
